# Multiscale Complexity Analysis of the Cardiac Control Identifies Asymptomatic and Symptomatic Patients in Long QT Syndrome Type 1

**DOI:** 10.1371/journal.pone.0093808

**Published:** 2014-04-04

**Authors:** Vlasta Bari, José F. Valencia, Montserrat Vallverdú, Giulia Girardengo, Andrea Marchi, Tito Bassani, Pere Caminal, Sergio Cerutti, Alfred L. George, Paul A. Brink, Lia Crotti, Peter J. Schwartz, Alberto Porta

**Affiliations:** 1 Gruppo Ospedaliero San Donato Foundation, Milan, Italy; 2 Department of Electronics, Information and Bioengineering, Politecnico di Milano, Milan, Italy; 3 Department of Electronic Engineering, Universidad de San Buenaventura, Cali, Colombia; 4 Department of Automatic Control, Center for Biomedical Engineering Research, Universitat Politècnica de Catalunya, Barcelona, Spain; 5 Center for Cardiac Arrhythmias of Genetic Origin, IRCCS Istituto Auxologico Italiano, Centro Diagnostico San Carlo, Milan, Italy; 6 Department of Anesthesia and Intensive Care, IRCCS Humanitas Clinical and Research Center, Rozzano, Italy; 7 IRCCS Humanitas Clinical and Research Center, Rozzano, Italy; 8 Department of Medicine and Pharmacology, Vanderbilt University, Nashville, Tennessee, United States of America; 9 Department of Internal Medicine, University of Stellenbosch, Stellenbosch, South Africa; 10 Institute of Human Genetics, Helmholtz Zentrum München, Neuherberg, Germany; 11 Department of Biomedical Sciences for Health, University of Milan, Milan, Italy; 12 IRCCS Galeazzi Orthopedic Institute, Milan, Italy; University of Adelaide, Australia

## Abstract

The study assesses complexity of the cardiac control directed to the sinus node and to ventricles in long QT syndrome type 1 (LQT1) patients with KCNQ1-A341V mutation. Complexity was assessed via refined multiscale entropy (RMSE) computed over the beat-to-beat variability series of heart period (HP) and QT interval. HP and QT interval were approximated respectively as the temporal distance between two consecutive R-wave peaks and between the R-wave apex and T-wave end. Both measures were automatically taken from 24-hour electrocardiographic Holter traces recorded during daily activities in non mutation carriers (NMCs, n = 14) and mutation carriers (MCs, n = 34) belonging to a South African LQT1 founder population. The MC group was divided into asymptomatic (ASYMP, n = 11) and symptomatic (SYMP, n = 23) patients according to the symptom severity. Analyses were carried out during daytime (DAY, from 2PM to 6PM) and nighttime (NIGHT, from 12PM to 4AM) off and on beta-adrenergic blockade (BBoff and BBon). We found that the complexity of the HP variability at short time scale was under vagal control, being significantly increased during NIGHT and BBon both in ASYMP and SYMP groups, while the complexity of both HP and QT variability at long time scales was under sympathetic control, being smaller during NIGHT and BBon in SYMP subjects. Complexity indexes at long time scales in ASYMP individuals were smaller than those in SYMP ones regardless of therapy (i.e. BBoff or BBon), thus suggesting that a reduced complexity of the sympathetic regulation is protective in ASYMP individuals. RMSE analysis of HP and QT interval variability derived from routine 24-hour electrocardiographic Holter recordings might provide additional insights into the physiology of the cardiac control and might be fruitfully exploited to improve risk stratification in LQT1 population.

## Introduction

The long QT syndrome is an inherited disease characterized by a prolonged ventricular repolarization duration, leading to a longer QT interval on the surface ECG [1], [2]. Long QT syndrome patients are at very high risk for life-threatening arrhythmias, such as torsades de pointes, and to sudden death [1], [2]. Several genetic mutations have been associated to long QT syndrome and one of the most common is the mutation on the KCNQ1 gene leading to long QT syndrome type 1 (LQT1). The occurrence of LQT1 symptoms is often precipitated by physiological conditions, such as physical or emotional stress, associated with an augmented sympathetic activity and an increased heart rate in a genotype-specific manner [3]. Although LQT1 is well coded in terms of genetic correlates, the same mutation can lead to totally different phenotypes or even to a complete absence of symptoms [2]. As a matter of fact, autonomic control can modulate the severity of LQT1. More specifically, the reactivity of the vagal control, estimated through the assessment of the cardiac baroreflex sensitivity and through the magnitude of the bradycardic response following exercise stress test, is helpful to divide a group carrying the same KCNQ1 mutation into symptomatic (SYMP) and asymptomatic (ASYMP) patients [4], [5]. Autonomic function might be modified in LQT1 patients and its assessment might provide the key for devising more powerful therapies and for improving risk stratification.

The spontaneous variations of heart period (HP) and QT interval provide indexes helpful to infer the state of the autonomic nervous system. For example, the power of HP variability in the high frequency (HF, from 0.15 to 0.4 Hz) band is a marker of vagal modulation directed to the sinus node [6], [7], being abolished by cholinergic blockade [8]. Conversely, QT variability is more under sympathetic control especially if the sympathetic drive is relevant [9]–[17]. This link was confirmed using a stimulus progressively increasing the sympathetic drive such as the graded head-up tilt test [18]–[20]: indeed, it was found that the amount of the QT changes in the low frequency (LF, from 0.04 to 0.15 Hz) band and the magnitude of the QT variations independent of HP changes increased with the relevance of the orthostatic challenge correlated with the inclination of the tilt table [10], [21]. Although linear indexes, such as the powers of the HP and QT variations in specific frequency bands, are largely utilized to infer the autonomic profile, there is an increasing amount of evidences that non linear indexes measuring the complexity of the cardiac control from HP and QT series might be more sensitive than the linear markers in the case of pathological conditions [22]. This propensity might come from their preserved relation with the state of the autonomic nervous system [23], their ability in accounting for non linear dynamics [22] and their link with the derangement of the cardiovascular control from a different perspective compared to the most frequently utilized autonomic markers based on the magnitude of HP and QT variations [24], [25].

We hypothesize that the complexity analysis of the HP and QT variability could be fruitfully exploited to characterize the LQT1 population and, more specifically, might contribute to differentiate patients with the same genetic defect but with completely different risks of cardiac events (i.e. ASYMP and SYMP groups). Unfortunately, since the HP and QT variability cannot be adequately modeled according to a sum of few periodic components, a single scale analysis might underestimate the complexity of the cardiac control and might have a limited power in distinguishing different groups, especially if they featured the same genotype. Conversely, the assessment of the HP and QT variability complexity as a function of the temporal scales might circumvent the limitations of single scale analysis [26].

The aim of this study was to perform the complexity analysis of the HP and QT variability to characterize cardiovascular control and favor the differentiation between ASYMP and SYMP patients. The availability of ASYMP and SYMP patients, all descendants from the same family originally settled in South Africa in 1690 [27] and carrying the same KCNQ1-A341V mutation, provides the unique possibility to study the adaptation process followed by the SYMP patients to limit the consequences of their genetic defect and the inner relation between genotype and phenotype [28]–[30]. As a matter of fact, the availability of a founder population such as this one offer the best chances for the identification of factors modifying the risk for life-threatening arrhythmias [31].

Complexity analysis was performed through multiscale entropy (MSE), first introduced by Costa et al [32] and subsequently refined by Valencia et al [33]. MSE allows the quantification of complexity of a time series as a function of the temporal scale, thus targeting specific control mechanisms concurring to the HP and QT regulation.

## Methods

### Generalities of MSE

MSE is a technique estimating the complexity of a time series x = {x(i), i = 1,…,N}, where i is the sample counter and N is the series length, via entropy rate at different time scales [32]. It consists of three steps performing: i) the elimination of the fast temporal scales via a low pass filtering procedure, thus obtaining the filtered series, x_f_ = {x_f_(i), i = 1,…,N}, that focuses the frequency range of interest; ii) the downsampling of x_f_ with a scale factor, τ, chosen according to the cutoff of the low pass filter exploited for canceling fast oscillations, thus obtaining the filtered downsampled series, x_f_^τ^ = {x_f_^τ^(j), j = 1,…,N/τ}; iii) the computation of an entropy rate over x_f_^τ^ as a function of τ. In this work we exploited a refined version of the MSE, the refined MSE (RMSE), devised to fix two biases present in the computation of the MSE [33].

### MSE and RMSE

The elimination of the fast temporal scales in MSE is carried out using a low pass finite impulse response filter performing the mean of τ samples (i.e. all coefficients of the filter are set to 1/τ) [32]. Since the frequency response of this filter is very poor being characterized by a slow roll-off of the main lobe, a large transition band and important side lobes, aliasing is not prevented when the filtered series is downsampled at a rate of one sample every τ. RMSE substitutes the low pass finite impulse response filter with a low pass Butterworth filter of order 6 having a cutoff frequency equal to 0.5/τ cycles/sample [33]. This filter has a flat response in the pass band, no side lobes in the stop band and a faster roll-off, thus being more efficient in limiting aliasing during downsampling. The low pass filtered series is downsampled at a rate of one sample every τ, thus reducing the total number of values from N to N/τ. The complexity of the low pass filtered series is estimated via the sample entropy [34]. Let us label the negative logarithm of the probability of finding two patterns of length L, x_f,L_^τ^(j) = [x_f_^τ^(j), x_f_^τ^(j-1),…, x_f_^τ^(j-L+1)] and x_f,L_^τ^(k) = [x_f_^τ^(k), x_f_^τ^(k-1),…, x_f_^τ^(k-L+1)] with L≤j,k≤N/τ at distance closer than a parameter r as Φ(L,r). The sample entropy is defined as the difference between Φ(L,r) and Φ(L-1,r), thus quantifying the probability that, if x_f,L-1_^τ^(j) and x_f,L-1_^τ^(k) are nearby in the embedding space of dimension L-1, they will remain nearby in the embedding space of dimension L [34]. r is usually referred to as tolerance for the calculation of the sample entropy and sets the level of coarse graining of the embedding space (i.e. patterns at distance closer than r cannot be distinguished as separated entities under the adopted level of discretization of the embedding space. In MSE r was set as a percentage of the standard deviation of x (usually the 15%) and it was kept constant as a function of τ [32]. Since the low pass filtering procedure reduces the standard deviation of the series, the region of the embedding space occupied by the patterns decreases more and more with τ and, consequently, the number of pairs of patterns becoming closer and closer increases with τ, thus leading to a decrease of the sample entropy. This complexity reduction is artificial because it is solely the effect of the decline of variance with τ. In RMSE r is set as a percentage of the standard deviation of x_f_^τ^, thus continuously updating r with τ [33].

### Study population, experimental protocol and data analysis

#### Ethics statement

The study adhered to the principles of the Declaration of Helsinki for medical research involving humans. It was approved by the ethical review boards of the Universities of Stellenbosch, Vanderbilt and Pavia. All probands and family members provided written informed consent for clinical and genetic evaluations, as approved by the ethical review boards of the Universities of Stellenbosch, Vanderbilt and Pavia. Written informed consent was obtained from the next of kin, caretakers or guardians on behalf of minors enrolled in the study. Full access to this database is available free of charge by contacting the corresponding author.

#### Study population and Holter recordings

Twelve lead 24-hour Holter recordings were acquired from 48 different individuals (age from 16 to 62, median = 41; 19 males) who were all heterozygous for the KCNQ1-A341V mutation and were members of a LQT1 founder population [27], [28]. The group was composed of 14 non mutation carriers (NMCs) (age from 19 to 56, median = 36.5; 6 males) and 34 mutation carriers (MCs). The MC group consisted of 11 ASYMP subjects (age from 24 to 62, median = 46; 4 males) and 23 SYMP individuals (age from 16 to 57, median = 39; 9 males).

While all NMC subjects were recorded only BBoff, 7 ASYMP and 22 SYMP subjects were recorded both on beta-blocker therapy (BBon) and off (BBoff). The remaining 5 MC individuals (i.e. 4 ASYMP and 1 SYMP) were acquired only BBoff. Beta-blocker therapy was quite homogeneous among the MC subjects with the majority of the patients (i.e. 86%) treated with propranolol.

The total number of recordings was 77: 14 traces from NMC recorded only BBoff, 58 recordings from 7 ASYMP and 22 SYMP acquired both BBoff and BBon, and 5 traces from 4 ASYMP and 1 SYMP recorded only BBoff. The majority of the recordings (i.e. 92%) were acquired using equipment from Mortara Instrument (Mortara Instrument Inc., Milwaukee, WI, USA) and the remaining subjects were studied using equipment from Ela Medical (Sorin Group, Arvada, CO, USA). Sampling rate was 180 Hz for Mortara and 200 Hz for Ela Medical recordings. Amplitude resolution was 6.25 and 10 μV for Mortara and Ela Medical recordings respectively. Analyses were carried out on the lead with the best signal-to-noise ratio. In the case of multiple recordings over the same subject, the procedure for lead selection prevented the choice of different leads over the same subject. Analyses were performed during daytime (DAY, from 2 PM to 6 PM) and nighttime (NIGHT, from 12 PM to 4 AM). The subjects were not asked to follow any specific behavioral procedure during the considered periods. Diaries of each subject were checked to ensure that the individual did not sleep during DAY period or remained awake during the NIGHT period. Sequences of 5000 consecutive HP and QT measures were randomly selected in the DAY and NIGHT periods.

#### Experimental protocol

We carried out three different comparisons. The first comparison checked the differences attributable to the genotype in the period most at risk for LQT1 patients (i.e. DAY) under the hypothesis that genotype affects the complexity of the cardiovascular control: we contrasted MC patients with NMC individuals BBoff during DAY (i.e. NMC-MC protocol). The second comparison evaluated the influence of the state of the autonomic nervous system on the considered parameters under the hypothesis that autonomic function can modulate the risk in LQT1 patients: we compared SYMP and ASYMP groups BBoff during DAY and NIGHT (i.e. DAY-NIGHT protocol). The third comparison evaluated the effect of beta-blocker therapy on the considered parameters in the period most at risk for LQT1 patients (i.e. DAY) under the hypothesis that beta-blocker therapy can affect the risk profile: we contrasted SYMP and ASYMP patients both BBoff and BBon during DAY (i.e. BBoff-BBon protocol).

#### Extraction of the HP and QT variabilities

HP was computed as the temporal distance between two consecutive R-wave apexes fixed with minimum jitters using parabolic interpolation. The QT interval was approximated as the time distance between R-wave peak and T-wave end. The T-wave end was located using a threshold set as a fraction of the maximal absolute first derivative computed on the T-wave downslope [35]. We make reference to [35] for ECG preprocessing procedures, baseline wandering removal and parameter settings for fiducial point delineation. The R-wave peak delimiting the i-th QT interval was the one defining the end of the i-th HP. All the parameters for R-wave apex and T-wave end recognition were continuously updated during the analysis and the detections were carefully checked. HP and QT series were not corrected except in case of premature ventricular contractions or evident arrhythmias. In these cases cubic spline interpolation was performed over the values to correct and the number of corrections was always lower than 5% of the total measures in the considered period of analysis.

#### HP and QT variability analyses

RMSE was computed over HP and QT series with τ ranging from 1 to 12. The time scales at τ = 1, τ ranging from 2 to 4, and τ ranging from 5 to 12 were defined as short, medium and long time scales respectively. According to the cutoff of the Butterworth filter (i.e. 0.5/τ cycles/sample) performing the analysis with τ = 1 is equivalent to traditional complexity analysis over the original unfiltered HP and QT series (i.e. from 0.0 to 0.5 cycles/beat). This analysis assesses the complexity of all temporal scales present in the HP and QT variability, being largely influenced by the fastest ones present in the original unfiltered series. Conversely, pooling together RMSE values assessed at medium time scales allowed the compact representation of the complexity at medium time scales: indeed, while varying τ from 2 to 4 the superior limit of the considered oscillations was reduced from 0.25 to 0.125 Hz with a HP mean of 1 s. This means that RMSE measures was calculated by getting rid of the contribution of the temporal scales above 0.25 Hz, by considering the contribution of respiratory oscillations (progressively canceled while increasing τ from 2 to 4) and by accounting for rhythms slower than the respiratory ones. Therefore, since complexity analysis is mainly influenced by the shortest time scale present in the series, this group of RMSE measures was primary affected by the contribution of oscillations present in the HF band. Finally, pooling together RMSE values assessed at long time scales allowed the compact representation of the complexity at long time scales: indeed, while varying τ from 5 to 12 the superior limit of the considered oscillations was reduced from 0.1 to 0.042 Hz with a HP mean of 1 s. As a consequence this group of RMSE measures was assessed after deleting fast periodicities including those in the HF band, thus accounting for the influences of rhythmicities in the LF band. According to this classification RMSE values were averaged over short, medium and long time scales and the mean value was labeled as RMSE_τ = 1_, RMSE_τ = 2–4_ and RMSE_τ = 5–12_ in the following.

#### Statistical analysis

We performed the paired t-test to check the significance of the difference between RMSE indexes derived from HP and QT series regardless of the considered group in each experimental protocol. If the normality test (Kolmogorov-Smirnov test) was not fulfilled, Wilcoxon signed rank test was utilized. One way analysis of variance (Holm-Sidak test for multiple comparisons), or Kruskal-Wallis one way analysis of variance on ranks (Dunn's method for multiple comparisons) when appropriate, was applied to check the significance of the differences between ASYMP, SYMP and NMC groups BBoff during DAY. Two way repeated measures analysis of variance (one factor repetition, Holm-Sidak test for multiple comparisons) was utilized to assess the significance of the differences between ASYMP and SYMP individuals BBoff in relation to the period of analysis (i.e. DAY and NIGHT) and between SYMP and ASYMP patients during DAY in relation to therapy (i.e. BBoff and BBon). Statistical analysis was carried out using a commercial statistical program (Sigmaplot, Systat Software, Inc, Chicago, IL, ver.11.0). A p<0.05 was always considered as significant.

## Results

### Time domain analysis

Table 1 shows mean and variance of the HP and QT series in the NMC-MC protocol. The HP mean, μ_HP_, was higher in ASYMP individuals than in NMC subjects. The HP variance, σ^2^_HP_, was comparable in all the populations. According to the pathology, the QT mean, μ_QT_, was longer in ASYMP and SYMP patients than in NMC individuals. The QT variance, σ^2^_QT_, separated ASYMP and SYMP patients with σ^2^_QT_ larger in ASYMP individuals than in SYMP ones.

**Table 1:** Time domain indexes derived from HP and QT series in the NMC-MC protocol.

	NMC (n = 14)	ASYMP (n = 11)	SYMP (n = 23)
μ_HP_ [ms]	697.6±100.6	847.9±143.8^§^	761.3±95.0
σ^2^_HP_ [ms^2^]	1195.8±711.8	1471.8±1048.1	1382.9±1000.6
μ_QT_ [ms]	317.6±39.2	422.2±51.7^§^	408.6±42.4^§^
σ^2^_QT_ [ms^2^]	186.0±243.3	271.4±212.0	115.4±48.8°

μ_HP_  =  HP mean; σ^2^_HP_  =  HP variance; μ_QT_  =  QT mean; σ^2^_QT_  =  QT variance; NMC  =  non mutation carrier group; ASYMP  =  asymptomatic group; SYMP  =  symptomatic group. Results are reported as mean ± standard deviation. The symbol ^§^ indicates p<0.05 versus NMC individuals. The symbol ° indicates p<0.05 versus ASYMP subjects.

Table 2 shows the same parameters reported in Tab.1 computed in the DAY-NIGHT protocol. In both ASYMP and SYMP patients μ_HP_ and μ_QT_ were longer during NIGHT than during DAY. In SYMP group σ^2^_HP_ increased during NIGHT, while in the ASYMP group σ^2^_QT_ decreased during NIGHT. Remarkably, during DAY σ^2^_QT_ was able to separate ASYMP subjects from SYMP ones. Indeed, during DAY σ^2^_QT_ of the SYMP group was smaller than that of the ASYMP one.

**Table 2:** Time domain indexes derived from HP and QT series in the DAY-NIGHT protocol.

	DAY		NIGHT	
	ASYMP (n = 11)	SYMP (n = 23)	ASYMP (n = 11)	SYMP (n = 23)
μ_HP_ [ms]	847.9±143.8	761.3±95.0	1022.6±136.3^*^	952.4±117.1^*^
σ^2^_HP_ [ms^2^]	1471.8±1048.1	1382.9±1000.6	1814.8±1619.9	2029.0±1897.3^*^
μ_QT_ [ms]	422.2±51.7	408.6±42.4	447.5±42.1^*^	445.3±31.2^*^
σ^2^_QT_ [ms^2^]	271.4±212.0	115.4±48.8^#^	95.6±75.1^*^	85.2±67.1

μ_HP_  =  HP mean; σ^2^_HP_  =  HP variance; μ_QT_  =  QT mean; σ^2^_QT_  =  QT variance; DAY  =  daytime; NIGHT  =  nighttime; ASYMP  =  asymptomatic group; SYMP  =  symptomatic group. Results are reported as mean ± standard deviation. The symbol * indicates p<0.05 within the same group (i.e. ASYMP or SYMP) versus DAY. The symbol ^#^ indicates p<0.05 within the same period of analysis (i.e. DAY or NIGHT) versus ASYMP subjects.

Table 3 shows the same parameters reported in Tabs.1,2 computed in the BBoff-BBon protocol. In both ASYMP and SYMP subjects beta-blocker therapy lengthened μ_HP_ but only μ_QT_ in SYMP patients was significantly increased. In the SYMP group σ^2^_HP_ was larger BBon than BBoff, while in ASYMP group beta-blocker therapy significantly reduced σ^2^_QT_. Given the same experimental condition (i.e. BBoff or BBon) μ_HP_, σ^2^_HP_ and σ^2^_QT_ were able to differentiate ASYMP individuals from SYMP ones. Indeed, BBon μ_HP_ was shorter and σ^2^_HP_ was larger in the SYMP group than in the ASYMP one and BBoff σ^2^_QT_ was smaller in the SYMP group than in the ASYMP one.

**Table 3:** Time domain indexes derived from HP and QT series in the BBoff-BBon protocol.

	BBoff		BBon	
	ASYMP (n = 7)	SYMP (n = 22)	ASYMP (n = 7)	SYMP (n = 22)
μ_HP_ [ms]	855.8±143.5	757.9±95.8	1038.2±176.0^*^	927.8±117.2^#,*^
σ^2^_HP_ [ms^2^]	1122.2±1014.7	1437.9±987.9	1581.0±1081.2	2667.9±1910.4^#,*^
μ_QT_ [ms]	424.0±57.6	406.5±42.1	426.7±58.0	429.8±29.3^*^
σ^2^_QT_ [ms^2^]	292.9±258.5	116.1±49.8^#^	110.9±105.3^*^	115.5±85.3

μ_HP_  =  HP mean; σ^2^_HP_  =  HP variance; μ_QT_  =  QT mean; σ^2^_QT_  =  QT variance; BBoff  =  off beta-blocker therapy; BBon  =  on beta-blocker therapy; ASYMP  =  asymptomatic group; SYMP  =  symptomatic group. Results are reported as mean ± standard deviation. The symbol ^*^ indicates p<0.05 within the same group (i.e. ASYMP or SYMP) versus BBoff. The symbol ^#^ indicates p<0.05 within the same therapy (i.e. BBoff or BBon) versus ASYMP subjects.

### Comparison between RMSE indexes derived from HP and QT series

**Figure 1:**
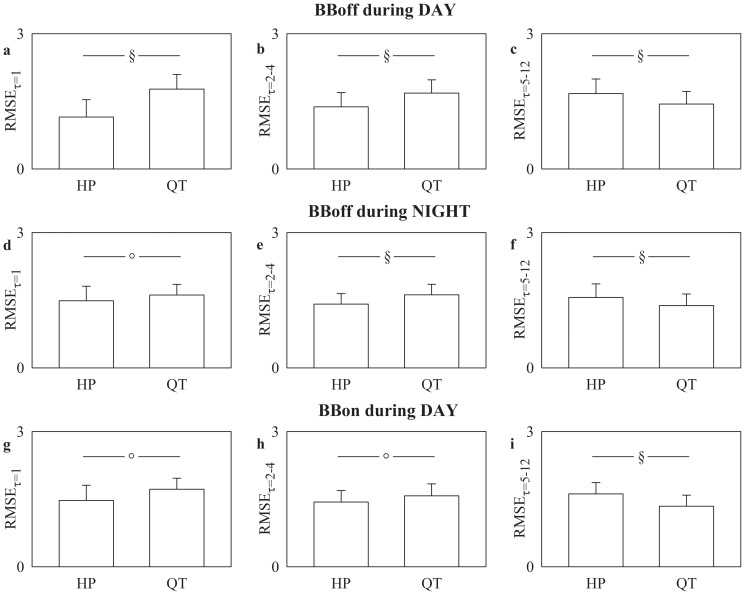
Comparison between RMSE indexes derived from HP and QT series. RMSE mean (plus standard deviation) assessed at short time scale (i.e. τ = 1), RMSE_τ = 1_ (a,d,g), at medium time scales (i.e. τ = 2–4), RMSE_τ = 2–4_ (b,e,h), and at long time scales (i.e. τ = 5–12), RMSE_τ = 5–12_, (c,f,i) is shown as a function of the time series (i.e. HP and QT). RMSE_τ = 1_, RMSE_τ = 2–4_, and RMSE_τ = 5–12_ were obtained by pooling RMSE values computed BBoff during DAY (a,b,c), BBoff during NIGHT in (d,e,f), and BBon during DAY (g,h,i), The symbols § and ° indicate a significant difference with p<0.001 and p<0.05 respectively.

Figure 1 shows RMSE mean (plus standard deviation) at short time scale (i.e. τ = 1, Figs.1a,d,g), RMSE_τ = 1_, at medium time scales (i.e. τ = 2–4 Figs.1b,e,h), RMSE_τ = 2–4_, and at long time scales (i.e. τ = 5–12, Figs.1c,f,i), RMSE_τ = 5–12_, as a function of the series (i.e. HP and QT). RMSE mean was computed by pooling together NMC, ASYMP and SYMP individuals BBoff during DAY in Figs.1a,b,c, ASYMP and SYMP subjects BBoff during NIGHT in Figs.1d,e,f, and ASYMP and SYMP individuals BBon during DAY in Figs.1g,h,i. BBoff during DAY RMSE_τ = 1_ and RMSE_τ = 2–4_ were significantly larger in the QT series than in the HP one. Conversely, the reverse situation was observed in the case of RMSE_τ = 5–12_. Similar results were found BBoff during NIGHT and BBon during DAY. However, the difference between RMSE_τ = 1_ indexes assessed over HP and QT series BBoff during NIGHT (Fig.1d) and BBon during DAY (Fig.1g) was less evident than BBoff during DAY (Fig.1a). RMSE_τ = 2–4_ exhibited the same trend (Figs.1b,e,h). The difference between RMSE indexes assessed over HP and QT series remained stable in the case of RMSE_τ = 5–12_ (Figs.1c,f,i).

### RMSE at short time scale (τ = 1) in NMC, ASYMP and SYMP groups

**Figure 2:**
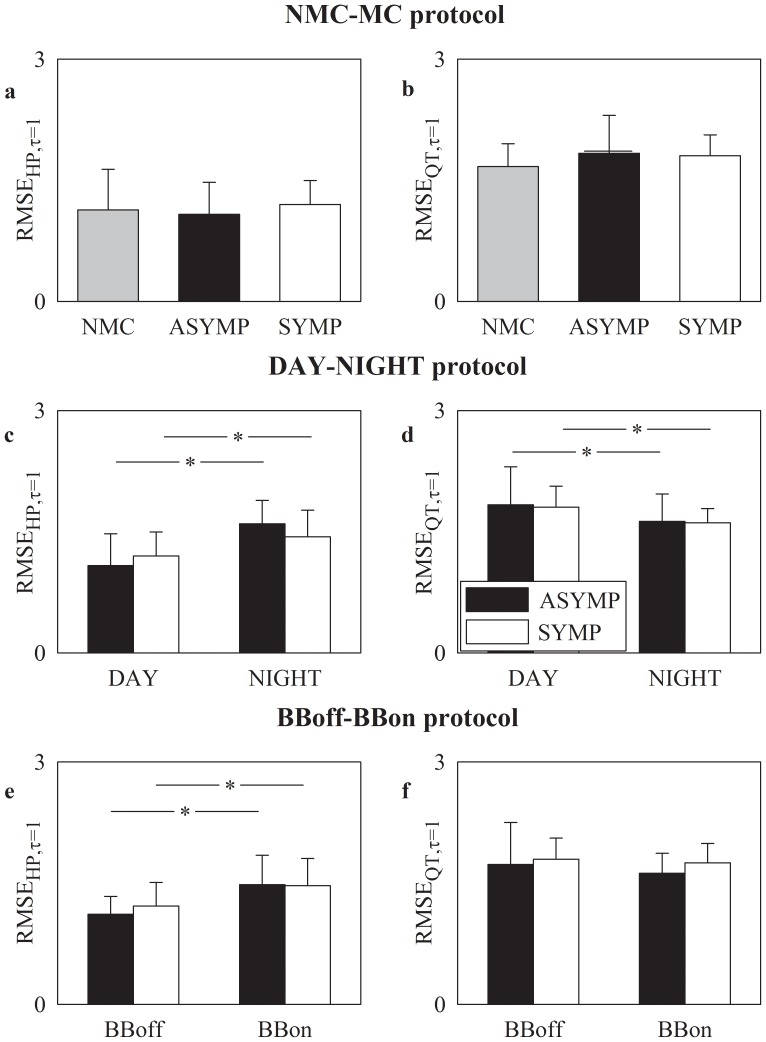
RMSE at short time scale over HP and QT series. RMSE mean (plus standard deviation) assessed at short time scale (i.e. τ = 1) over HP series, RMSE_HP,τ = 1_ (a,c,e), and QT series, RMSE_QT,τ = 1_ (b,d,f), is shown as a function of the experimental protocol. RMSE_HP,τ = 1_ and RMSE_QT,τ = 1_ are depicted as a function of the group of subjects in the NMC-MC protocol in (a) and (b) respectively, as a function of the period of analysis in the DAY-NIGHT protocol in (c) and (d) respectively, and as a function of the therapy in the BBoff-BBon protocol in (e) and (f) respectively. The gray, dark and white bars are relevant to NMC, ASYMP and SYMP individuals, respectively. The symbols * indicates a significant difference between experimental conditions (i.e. DAY versus NIGHT, BBoff versus BBon) within the same group with p<0.05.

Figure 2 shows RMSE mean (plus standard deviation) at short time scale (i.e. τ = 1) as derived from HP and QT series (i.e. RMSE_HP,τ = 1_ and RMSE_QT,τ = 1_) in Figs.2a,c,e and Figs.2b,d,f respectively. RMSE_τ = 1_ was unable to separate groups (i.e. NMC, ASYMP and SYMP). This conclusion held for any series (i.e. HP or QT) and for any protocol (i.e. NMC-MC, DAY-NIGHT or BBoff-BBon). In both ASYMP and SYMP groups RMSE_HP,τ = 1_ significantly increased during NIGHT compared to DAY (Fig.2c) and BBon compared to BBoff (Fig.2e). While RMSE_QT,τ = 1_ significantly decreased during NIGHT compared to DAY in both ASYMP and SYMP patients (Fig.2d), beta-blocker therapy did not modify RMSE_QT,τ = 1_ (Fig.2f).

### RMSE at medium time scales (τ = 2–4) in NMC, ASYMP and SYMP groups)

**Figure 3:**
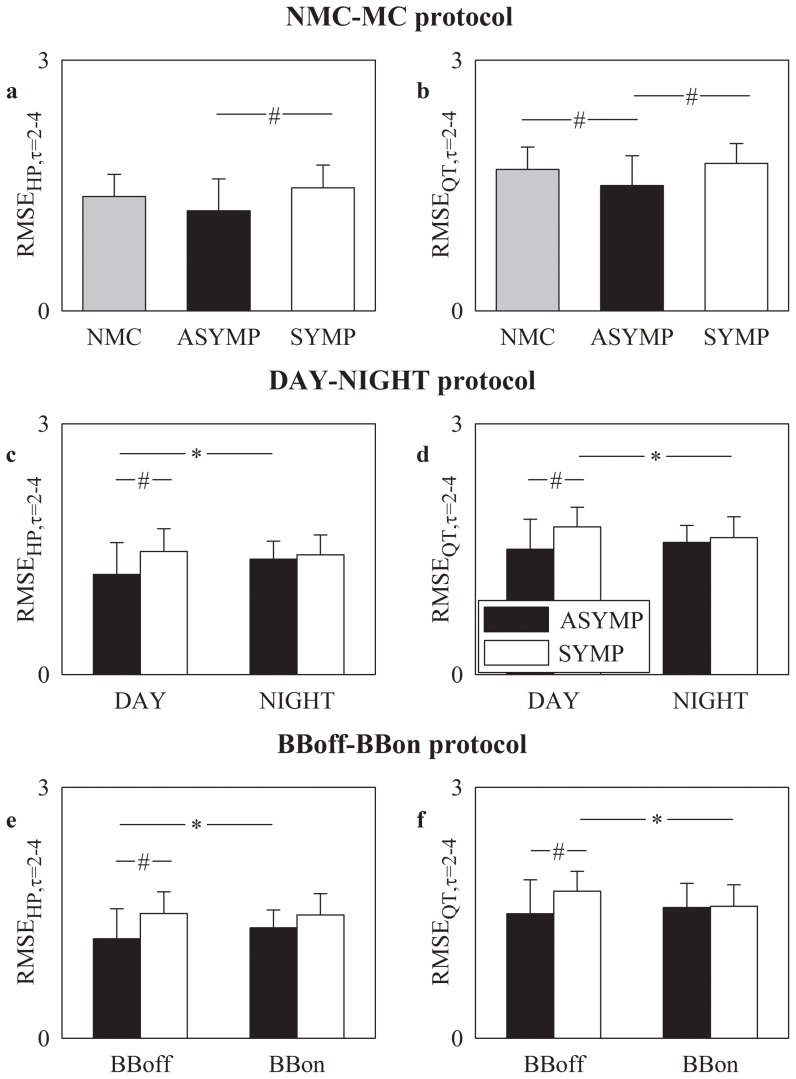
RMSE at medium time scales over HP and QT series. RMSE mean (plus standard deviation) assessed at medium time scales (i.e. τ = 2–4) over HP series, RMSE_HP,τ = 2–4_ (a,c,e), and QT series, RMSE_QT,τ = 2–4_ (b,d,f), is shown as a function of the experimental protocol. RMSE_HP,τ = 2–4_ and RMSE_QT,τ = 2–4_ are depicted as a function of the group of subjects in the NMC-MC protocol in (a) and (b) respectively, as a function of the period of analysis in the DAY-NIGHT protocol in (c) and (d) respectively, and as a function of the therapy in the BBoff-BBon protocol in (e) and (f) respectively. The gray, dark and white bars are relevant to NMC, ASYMP and SYMP individuals, respectively. The symbol * indicates a significant difference between experimental conditions (i.e. DAY versus NIGHT, BBoff versus BBon) within the same group with p<0.05. The symbol ^#^ indicates a significant difference between groups within the same experimental conditions (i.e. DAY, NIGHT, BBoff or BBon) with p<0.05.

Figure 3 shows RMSE mean (plus standard deviation) at medium time scales (i.e. τ = 2–4) as derived from HP and QT series (i.e. RMSE_HP,τ = 2–4_ and RMSE_QT,τ = 2–4_) in Figs.3a,c,e and Figs.3b,d,f respectively. In the NMC-MC protocol RMSE_HP,τ = 2–4_ (Fig.3a) and RMSE_QT,τ = 2–4_ (Fig.3b) were able to separate the ASYMP group from the SYMP one with both indexes smaller in the ASYMP group. RMSE_QT,τ = 2–4_ differentiated the ASYMP group from the NMC one as well, being RMSE_QT,τ = 2–4_ in the ASYMP group smaller than in NMC one (Fig.3b). In the DAY-NIGHT protocol (Figs.3c,d) and in the BBoff-BBon protocol (Figs.3e,f) RMSE_HP,τ = 2–4_ and RMSE_QT,τ = 2–4_ separated the ASYMP group from the SYMP one only during DAY with both RMSE_HP,τ = 2–4_ and RMSE_QT,τ = 2–4_ higher in SYMP patients than in ASYMP group. DAY-NIGHT variations and the effect of the therapy were observable only in RMSE_HP,τ = 2–4_ in ASYMP group with RMSE_HP,τ = 2–4_ significantly increased during NIGHT (Fig.3c) and BBon (Fig.3e) and only in RMSE_QT,τ = 2–4_ in SYMP group with RMSE_QT,τ = 2–4_ significantly decreased during NIGHT (Fig.3d) and BBon (Fig.3f).

### RMSE at long time scales (τ = 5–12) in NMC, ASYMP and SYMP groups

**Figure 4:**
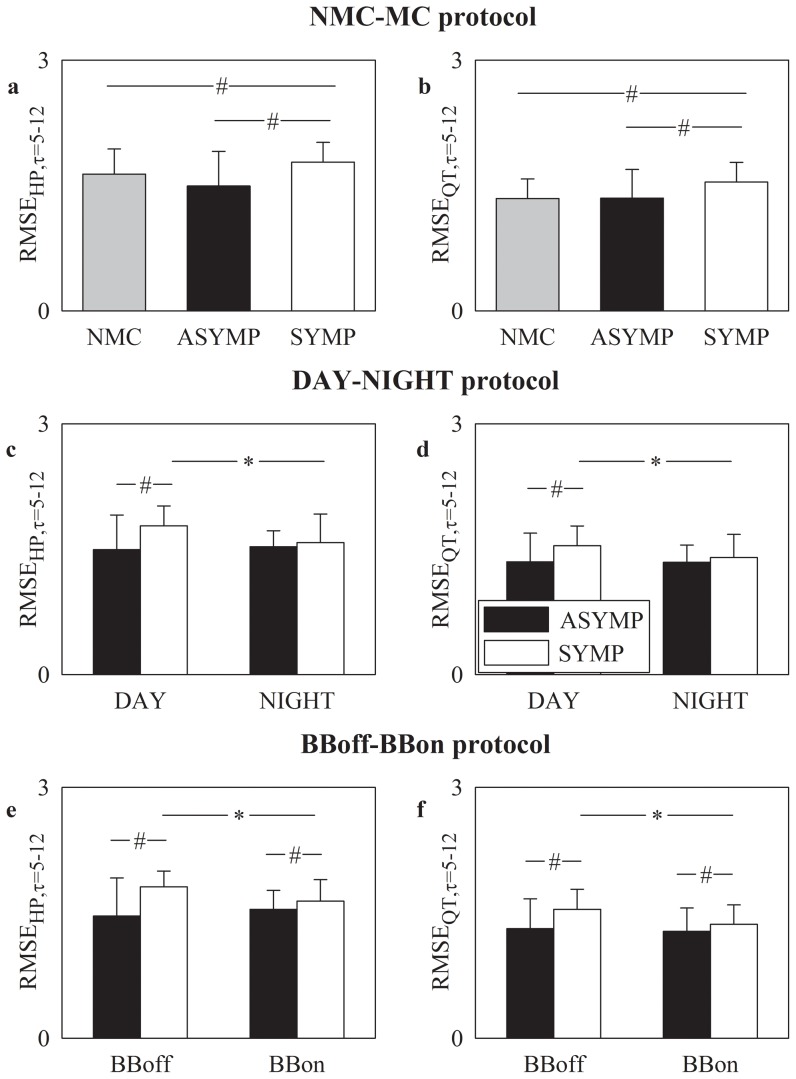
RMSE at long time scales over HP and QT series. RMSE mean (plus standard deviation) assessed at long time scales (i.e. τ = 5–12) over HP series, RMSE_HP,τ = 5–12_ (a,c,e), and QT series, RMSE_QT,τ = 5–12_ (b,d,f), is shown as a function of the experimental protocol. RMSE_HP,τ = 5–12_ and RMSE_QT,τ = 5–12_ are depicted as a function of the group of subjects in the NMC-MC protocol in (a) and (b) respectively, as a function of the period of analysis in the DAY-NIGHT protocol in (c) and (d) respectively, and as a function of the therapy in the BBoff-BBon protocol in (e) and (f) respectively. The gray, dark and white bars are relevant to NMC, ASYMP and SYMP individuals, respectively. The symbol * indicates a significant difference between experimental conditions (i.e. DAY versus NIGHT, BBoff versus BBon) within the same group with p<0.05. The symbol ^#^ indicates a significant difference between groups within the same experimental conditions (i.e. DAY, NIGHT, BBoff or BBon) with p<0.05.

Figure 4 shows RMSE mean (plus standard deviation) at long time scales (i.e. τ = 5–12) as derived from HP and QT series (i.e. RMSE_HP,τ = 5–12_ and RMSE_QT,τ = 5–12_) in Figs.4a,c,e and Figs.4b,d,f respectively. In the NMC-MC protocol RMSE_HP,τ = 5–12_ and RMSE_QT,τ = 5–12_ were able to separate the SYMP group from NMC and ASYMP individuals with RMSE_HP,τ = 5–12_ and RMSE_QT,τ = 5–12_ in the SYMP group larger than those in the NMC and ASYMP ones (Figs.4a,b). In the DAY-NIGHT protocol RMSE_HP,τ = 5–12_ (Fig.4c) and RMSE_QT,τ = 5–12_ (Fig.4d) separated the ASYMP group from the SYMP one only during DAY with RMSE_HP,τ = 5–12_ and RMSE_QT,τ = 5–12_ larger in SYMP individuals. Significant DAY-NIGHT variations were observed only in SYMP individuals (Figs.4c,d) with both RMSE_HP,τ = 5–12_ and RMSE_QT,τ = 5–12_ smaller during NIGHT (Figs.4c,d). In the BBoff-BBon protocol RMSE_HP,τ = 5–12_ and RMSE_QT,τ = 5–12_ distinguished ASYMP individuals from SYMP ones both BBoff and BBon with both RMSE_HP,τ = 5–12_ and RMSE_QT,τ = 5–12_ larger in SYMP individuals (Figs.4e,f). As to the effect of the beta-blocker therapy, it was visible only in SYMP patients over RMSE_HP,τ = 5–12_ and RMSE_QT,τ = 5–12_ (Fig.4e,f). The effect of the therapy was to reduce both RMSE_HP,τ = 5–12_ (Fig.4e) and RMSE_QT,τ = 5–12_ (Fig.4f).

## Discussion

The major findings of the study can be summarized as follows.

First, we confirmed the protective role of having longer HP in LQT1 syndrome [4] and the larger vagal reactivity of SYMP subjects [4], [5] as indicated by a larger HP variance during NIGHT. As a new finding the increased vagal reactivity appeared to be associated with a reduced sympathetic control in the SYMP group as indicated by the low QT variance during DAY.

Second, HP and QT variability were characterized by different levels of complexity and this difference was linked to the activity of the autonomic nervous system. At short time scale the sympathetic branch of the autonomic nervous system played a central role in keeping high the complexity of the QT series, while the vagal branch of the autonomic nervous system was involved in keeping high the complexity of the HP series. At long time scales sympathetic nervous system was involved in modulating the complexity of both HP and QT series even though the contribution to the complexity of the HP and QT variability was different.

Third, complexity indexes derived from HP and QT series at short time scales could not to differentiate the groups under scrutiny, while those at medium and long time scales could. More specifically, complexity markers at medium and long time scales assessed over both HP and QT series increased in SYMP group compared to the ASYMP one, thus suggesting a higher complexity of the sympathetic control in SYMP group.

Fourth, complexity indexes derived from HP and QT series at long time scales exhibited a tendency toward a reduction overnight and under beta-adrenergic therapy, thus indicating a beneficial effect of the therapy and a reduced cardiac risk while sleeping.

We conclude that multiscale complexity analysis is helpful in identifying LQT1 patients with different cardiac risks and insightful in describing the cardiovascular control of LQT1 patients.

### Time domain analysis of HP and QT variability

We confirmed that ASYMP patients had longer HP [4]. This characteristic can be considered as a protective factor because, in presence of a relatively immutable duration of the T-wave, it decreases the likelihood that a new ventricular depolarization could occur in the wrong phase of the T wave. This observation substantiates the protective effect of the beta-blocker therapy in LQT1 patients because it leads to a HP lengthening [36]. Since the HP prolongation induced by the beta-blocker therapy was larger in ASYMP patients than in SYMP ones, it could be conjectured that the beta-blocker treatment is more effective in ASYMP individuals than in SYMP one. We observed that HP mean was longer during NIGHT than during DAY in both ASYMP and SYMP group. Since having a longer HP might be safer in LQT1 patients [4], this finding might explain why LQT1 individuals are less at risk during NIGHT than during DAY [3]. According to the LQT1 phenotype QT mean was longer in ASYMP and SYMP groups compared to the NMC one. Circadian rhythm of the QT mean was preserved in both ASYMP and SYMP, thus suggesting that the positive relation linking HP to QT was maintained in LQT1 patients. In SYMP patients the power of the HP variability increased during NIGHT in absence of therapy, as a likely result of a vagal enhancement [6], [8], and BBon during DAY, as a likely result of the beta-adrenergic blockade [37]. This result suggests a more reactive vagal control in SYMP patients [4], [5]. This observation is in agreement with the finding that the cardiac baroreflex sensitivity, another index of vagal modulation, was increased in SYMP patients [4] and that SYMP patients were characterized by an exaggerated bradycardia following an exercise stress test [5]. As a novel finding the ASYMP patients were characterized by a higher QT variance compared to the SYMP ones during DAY in absence of therapy, thus suggesting that a higher sympathetic modulation might be a protective factor in ASYMP patients because it is helpful in limiting the vagal control and its responsiveness to challenges. The observed reduction of the QT variance in ASYMP subjects BBoff during NIGHT and BBon during DAY confirms that the amount of the QT variability is under sympathetic control [9]–[12], [16], [17], [21], [38].

### Complexity analysis of HP and QT series provides non redundant information in LQT1 patients

In LQT1 population we confirm that QT complexity indexes provide different information from the HP complexity ones [26]. Differences depend on the temporal scales under scrutiny. While at short and medium temporal scales the complexity of the QT series was larger than that of the HP series [39], [40], the reverse situation was found at long temporal scales [26]. This result was independent of the period of analysis (i.e. DAY or NIGHT) and of the therapy (i.e. BBoff or BBon). The larger complexity of QT series at short and medium temporal scales suggests that the QT variability cannot be considered merely the consequence of the HP changes inducing QT variations through the QT-HP relation [41]. Inputs modifying QT interval and its variability independently of HP changes [10], [13], [42]–[44] tend to increase the complexity of the QT series compared to that of the HP one. We suggest that the complexity of the QT series at short and medium time scales was kept high by QT dynamics unrelated to HP changes. Since these inputs increase during the sympathetic activation induced by an orthostatic challenge [10] and mental stress [13], it is not surprising to find out that the difference between QT and HP complexity tends to decrease during NIGHT and in presence of beta-blocker therapy. Also the smaller complexity of the QT series at long time scales compared to that of the HP series is incompatible with an all-pass QT-HP transfer function. It might be hypothesized that inputs at long time scales targeting the sinus node cannot reach ventricles, thus suggesting that the control of the ventricles at long time scales is much simpler than that of the sinus node.

### Link between complexity indexes at short time scale and autonomic regulation in LQT1 population

Complexity of the HP series at short temporal scale is mainly under vagal control. Indeed, it decreased significantly after complete cholinergic blockade induced by a high dose administration of atropine [45] and during vagal withdrawal induced by head-up tilt [23] or active standing [46]. The present study confirms the link between complexity of the HP series at short time scale and vagal control: indeed, complexity of the HP series increased during NIGHT [47], as a likely consequence of the increased importance of vagal modulation, and in presence of beta-blocker therapy, as a likely consequence of the sympathetic blockade leading to an augmented respiratory sinus arrhythmia [37]. The increase was significant in both SYMP and ASYMP patients, thus suggesting that the circadian rhythm was preserved and effects of beta-blocker therapy were evident in both groups.

Fewer studies tried to establish an association between complexity of QT series at short time scale and autonomic modulation. Some studies suggested that an augmented complexity of QT series could be interpreted as a marker of a higher sympathetic drive [40], [48]. The present study detected a reduction of complexity of the QT series at short time scale during NIGHT. Since QT series is mainly under sympathetic control [9]–[12], [16], [17], [21], [38], this tendency suggests a simplification of the cardiac control directed to ventricles during NIGHT, as a likely result of the vagal enhancement. However, since beta-blocker therapy did not affect the complexity of the QT series at short time scale, the association between the complexity of the QT series at short time scale and sympathetic control appears to be weak, thus prompting for the search of this association at time scales more compatible with the sluggishness of the sympathetic control.

### Link between complexity indexes at medium and long time scales and autonomic regulation in LQT1 population

The physiological correlates of the complexity of the HP series assessed at medium and long temporal scales are not completely identified. Since complexity indexes at medium time scales assessed from the QT series and at long time scales estimated from both HP and QT series decreased during sympathetic withdrawal occurring during NIGHT and after beta-adrenergic blockade, we suggest that these parameters are under sympathetic control. This observation is in agreement with the finding that complexity indexes at long temporal scales increased during the sympathetic activation induced by active standing [46]. It is worth noting that, although complexity indexes at medium and long time scales exhibited similar trends, the ones at long time scales appeared to be more powerful in suggesting the simplification of the sympathetic control during NIGHT and due to the beta-blocker treatment. As a consequence, we recommend the sole calculation of complexity indexes at long time scales in future applications aiming at extracting indexes linked to the sympathetic function.

### Complexity analysis at short time scale was unable to distinguish SYMP patients from ASYMP ones in LQT1 population

One of the most important finding of this study is that the most commonly utilized index of complexity based on entropy (i.e. RMSE at short time scale) [34] failed to differentiate MC individuals from NMC patients and to separate MC individuals into SYMP and ASYMP subjects. This finding was robust because it did not depend on the considered variability series (i.e. HP or QT series) and on the experimental protocol (i.e. NMC-MC, DAY-NIGHT and BBoff-BBon protocols). This conclusion substantiates the need of adopting the MSE approach in this specific study. A likely explanation of this disappointing result might be the low temporal resolution of HP and QT measures derived from a historical database of Holter recordings [27], [28]. This low temporal resolution might increase the corrupting influence of noise, especially at high frequencies (i.e. close to superior limit of the HF band), thus limiting the information content of faster temporal scales and reducing the statistical power of RMSE indexes at short time scale.

### Complexity analysis at medium and long time scales did distinguish SYMP patients from ASYMP ones in LQT1 population

At difference with complexity indexes at short time scale, the ones at medium and long time scales did distinguish ASYMP individuals from SYMP ones especially during DAY. Complexity of the cardiac control at medium and long time scales was larger in SYMP subjects than in ASYMP individuals. Since the increase was observed in the case of both indexes at medium and long time scales, it cannot be ascribed to oscillations in the HF band but to slower temporal scales (i.e. in the LF band or even below the inferior limit of the LF band). Differences in vagal control between SYMP and ASYMP individuals cannot fully explain this differentiation. Indeed, if vagal control was responsible for the increase of the complexity of the cardiac control at medium and long time scales, the complexity indexes at medium and long time scales derived from the QT series would have remained unchanged because QT variability is more responsive to sympathetic control and largely unaffected by the vagal control [21]. Therefore, data suggest that in SYMP patients the sympathetic control impinging both sinus node and ventricles is more complex than that of ASYMP subjects. Since ASYMP subjects are characterized by a lower probability of cardiac events, we suggest that a smaller complexity of the cardiovascular control is protective. In addition, since complexity indexes assessed from the QT series at medium time scales and assessed from HP and QT series at long time scales decreased during NIGHT and due to the beta-blocker therapy in SYMP subjects, complexity analysis confirms that NIGHT is a safer period for LQT1 patients and beta-blocker therapy is beneficial. This conclusion is supported also by the reduced differences between ASYMP and SYMP patients during NIGHT and after beta-blocker therapy.

## Conclusions

RMSE was applied to assess the complexity of the cardiac control directed to sinus node and to ventricles in NMC and MC individuals all being descendants of the same South African family. The study demonstrates the different information carried by markers of complexity derived from the HP and QT variability and the importance of assessing complexity as a function of the temporal scales in LQT1 population. Indeed, while the complexity of the HP series at short time scale was under vagal control, the complexity of the HP and QT variability at long time scales was under sympathetic control. In addition, the study proves the clinical relevance of the complexity analysis of the cardiac control in LQT1 patients. Indeed, the detected ability of the complexity indexes at long time scales to separate the ASYMP group from the SYMP one suggests that the complexity of the sympathetic control acts as an arrhythmic risk modifier in LQT1 patients with individuals characterized a larger complexity having a higher probability of belonging to the SYMP group. Remarkably, the separation between ASYMP and SYMP groups did not necessitate any complex procedure, being achieved over routine 24-hour Holter electrocardiographic recordings. Since conclusions were achieved over electrocardiographic traces with low temporal resolution, the proposed analysis is suitable for retrospective applications to historical databases. In addition, findings support the suitability of the most common LQT1 therapy based on beta-adrenergic blockade in limiting the arrhythmic risk in LQT1 patients. Indeed, given that a high complexity of the sympathetic control is a risk factor in LQT1 patients, beta-adrenergic therapy can successfully limit it and reduce the differences between ASYMP and SYMP subjects.
